# Identification and Analysis of the Terpene Synthases (TPS) Gene Family in Camellia Based on Pan-Genome

**DOI:** 10.3390/genes17010094

**Published:** 2026-01-17

**Authors:** Renjie Yin, Haibin Liu, Shanyuanrui Lin, Zhuolin Li, Linna Ma, Peng Liu

**Affiliations:** College of Life Sciences, North China University of Science and Technology, Tangshan 063210, China; 13856344078@163.com (R.Y.); liuhaibinhappy1013@126.com (H.L.); xiulianaiqing666@163.com (S.L.); 15848384620@163.com (Z.L.); malinna600912@163.com (L.M.)

**Keywords:** terpene synthase (TPS), pan-genome, copy number variation (CNV), tea domestication

## Abstract

Terpenes are major determinants of tea aroma, and terpene synthases (TPSs) catalyze key steps in terpenoid biosynthesis. To capture gene-family variation beyond a single reference, we performed a pan-genome–based analysis of TPS genes across nine *Camellia* genomes (three wild tea relatives and six cultivated *Camellia sinensis* accessions) and integrated pan-transcriptome profiling across eight tissues. We identified 381 TPS genes; wild species contained more TPSs than cultivated accessions (mean 58.3 vs. 34.3), suggesting a putative contraction. Phylogenetic analysis with *Arabidopsis* TPSs classified *Camellia* TPSs into five subfamilies, dominated by TPS-b (149) and TPS-a (140), whereas TPS-c was rare (8). Gene-structure and physicochemical analyses revealed marked subfamily divergence, with TPS-c showing highly conserved coding-region length. Orthology clustering assigned 355 TPSs to 19 orthogroups, including five core groups (190 genes, 53.5%) and 14 dispensable groups (165 genes, 46.5%); core/non-core status was significantly associated with subfamily composition. Tandem and proximal duplication contributed most to TPS expansion (29.4% and 29.1%), and all orthogroups exhibited copy-number variation, with pronounced lineage-specific expansions. Ka/Ks analyses indicated pervasive purifying selection (median 0.516) but heterogeneous constraints among subfamilies. Finally, cultivated tea showed higher TPS expression in most tissues, especially mature leaf and stem, and TPS-g displayed the broadest and strongest expression. Together, these results provide a pan-genome resource for *Camellia* TPSs and highlight how domestication, duplication, and CNV shape terpene-related genetic diversity.

## 1. Introduction

Tea plant (*Camellia sinensis*) is an important specialty cash crop in China, with an industrial chain covering cultivation, processing, and consumption, providing strong support for the agricultural economy in major producing regions [1]. Aroma is shaped by multiple classes of volatile metabolites, among which terpenoid volatiles account for a substantial proportion of characteristic floral, fruity, and fresh aroma notes and are closely associated with differences and stability of tea flavor [2]. Terpene synthases (terpene synthase, TPS) catalyze the conversion of universal isoprenoid precursors into diverse terpene skeletons and represent a key enzyme family linking upstream precursor supply to downstream diversification of aroma products [3]. Therefore, systematically dissecting the composition, evolution, and expression characteristics of the tea TPS family is important for understanding tea aroma formation and for supporting quality improvement and molecular breeding.

The TPS family shares a common sequence and structural feature characterized by a “highly conserved catalytic core + variable N-terminal region” [4]. The catalytic core is generally composed of a C-terminal terpene synthase domain and contains conserved motifs closely related to metal-ion binding and catalytic cascades, most typically the DDxxD and NSE/DTE (e.g., NDxxSxxxE/DTE) motifs, which coordinate Mg^2+^/Mn^2+^ and trigger substrate ionization and carbocation cascade reactions to generate diverse terpene skeletons [1,5]. Some TPSs (especially branches associated with monoterpenes) also frequently harbor conserved segments such as RRx8W, which are implicated in the initiation/stabilization of monoterpene cyclization [6,7]. In contrast, some members related to diterpene pathways (e.g., CPS-like proteins in the TPS-c clade) contain class II cyclization-related motifs such as DXDD, reflecting mechanistic divergence [8,9]. Beyond catalytic sites, TPS proteins differ markedly in N-terminal length, the presence/absence of plastid transit peptides, and insertion/deletion events; these changes can affect subcellular localization (plastid/cytosol) and substrate accessibility (GPP/FPP/GGPP), and are further associated with product-spectrum diversity and tissue-specific regulation [10,11]. Terrestrial plant TPS families are typically divided into multiple subfamilies (commonly TPS-a, TPS-b, TPS-c, TPS-e/f, and TPS-g, and expanded to TPS-a–TPS-h in broader lineages), and different subfamilies show relatively stable trends of functional specialization in substrate utilization and product types: TPS-a has been frequently associated with sesquiterpene biosynthesis, TPS-b and TPS-g are more closely linked to monoterpene/volatile formation, whereas TPS-c and TPS-e/f are often related to diterpene biosynthesis and growth/developmental pathways such as gibberellins [12].

To date, TPS families have been identified and analyzed in multiple important crops and plants, including *Arabidopsis thaliana* [13], tomato [14], rice [15], grapevine [16], wild mint [17], celery [18], and eucalyptus [19]. However, conventional studies largely rely on a single reference genome and thus cannot comprehensively capture gene presence/absence variation and copy number variation (CNV) that are widespread within and among species, potentially underestimating the true family size and compositional diversity. With advances in sequencing and high-quality assemblies and the release of multi-genome resources for *Camellia*, the pangenome framework provides new opportunities to resolve gene families at a multi-genome scale [20,21,22]. In this study, we integrated representative *Camellia* genome resources to systematically identify TPS genes and construct a phylogenetic classification, and we further performed comprehensive analyses of gene structure and physicochemical properties, orthogroup clustering and core/variable composition, duplication types and CNV features, selection-pressure patterns, and multi-tissue expression profiles, providing resources and clues for understanding the evolutionary dynamics of the *Camellia* TPS family and its potential roles in aroma-related metabolism.

## 2. Materials and Methods

### 2.1. Data Sources

Protein sequences, coding DNA sequences (CDS), and transcriptome expression matrices (TPM) for nine *Camellia* species were downloaded from the Tea Plant Information Archive (TPIA; https://tpia.teaplants.cn/ (accessed on 1 January 2026)) [23]. The TPS domain HMM profiles (PF01397 and PF03936) were obtained from the InterPro database (https://www.ebi.ac.uk/ (accessed on 1 January 2026)). Based on domestication history, the nine *Camellia* accessions were classified into wild and cultivated tea plants.

### 2.2. Tps Family Identification and Physicochemical Property Analysis

We used a Python v3.14.2 script to extract the longest isoform for each gene. TPS genes were first identified using PF01397.hmm and PF03936.hmm with HMMER3.0 [24], with an E-value threshold ≤ 1 × 10^−5^. In addition, the 33 TPS members from *A. thaliana* were used as references to perform BLASTP searches against the other genomes (E-value ≤ 1 × 10^−5^). The candidate sets from HMMER and BLASTP were merged (union). All candidates were then annotated using InterProScan5.18-57.0 with all available databases enabled [25]. Finally, a Python script was used to retain genes containing both TPS domains as the final TPS set, and gene numbers were summarized [26]. Physicochemical properties of all TPS proteins were predicted using the Protein Parameter Calc module in TBtools-II [27].

### 2.3. Phylogenetic Analysis

Multiple sequence alignment of TPS protein sequences from the nine *Camellia* genomes was performed using MAFFT (v7.475) [28]. We then trimmed the multiple sequence alignment using trimAl (v1.5.0) with default settings prior to phylogenetic tree reconstruction. A maximum-likelihood phylogenetic tree was inferred using IQ-TREE (v2.4.0), and ModelFinder automatically selected JTT + R6 as the best-fit substitution model [29], with the best-fit model automatically selected and 1000 bootstrap replicates. Clades were then defined according to tree topology and previous reports. The phylogenetic tree was visualized and annotated using iTOLv7.3 (https://itol.embl.de (accessed on 1 January 2026)) [30].

### 2.4. Pangenome Analysis of the Tps Gene Family

Orthogroup inference was performed for the identified TPS genes from the nine *Camellia* genomes using OrthoFinder (v2.5.4) (orthofinder -t 64 -a 64 -M msa -S diamond -A mafft -T fasttree) [31]. TPS genes were classified as core (present in all genomes) or non-core (absent in at least one genome).

### 2.5. Gene Duplication-Type Analysis

Protein sequences from the nine *Camellia* genomes were aligned using DIAMOND v2.1.16 [32] with an E-value threshold ≤ 1 × 10^−5^. Gene duplication types were then assigned using the duplicate_gene_classifier program in MCScanX [33].

### 2.6. Selection Pressure Analysis

Using the CDS and protein-coding sequence files of the nine *Camellia* species as inputs, Ka, Ks, and Ka/Ks values for homologous pairs within all orthogroups (OGGs) were calculated with the Simple Ka/Ks Calculator module in TBtools-II.

### 2.7. Data Processing

All statistical tests were performed using custom Python scripts (Python v3.14.2; NumPy v2.4.1, pandas v2.3.3, SciPy v1.17.0, statsmodels v0.14.6, and openpyxl v3.1.5), and all figures were generated using R scripts (R v4.5.2; ggplot2 v4.0.1, dplyr v1.1.4, tidyr v1.3.2, and readr v2.1.6).

## 3. Results

### 3.1. Identification and Phylogenetic Analysis of Tps Family Genes

**Figure 1:**
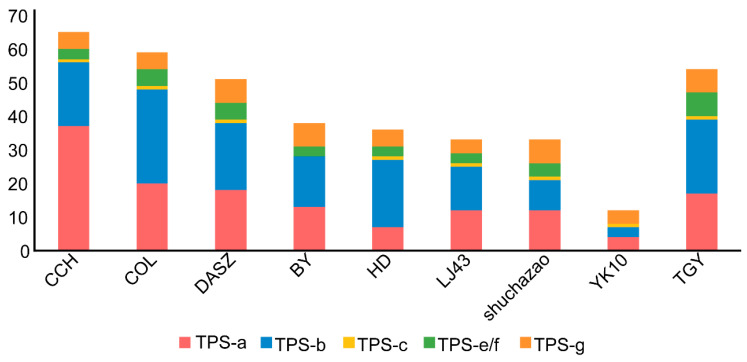
Numbers of TPS family genes from nine *Camellia* species in each TPS subfamily; the *x*-axis shows the abbreviations of the *Camellia* species (defined in Table S2).

We downloaded nine genomes from the genus *Camellia*, including three wild tea relatives (*Camellia taliensis*, *Camellia oleifera*, and *Camellia chekiangoleosa*) and six commonly cultivated accessions within *C. sinensis*, namely Shuchazao, Biyun, Huangdan, Tieguanyin, Longjing43, and Yunkang10. In total, 381 TPS family genes were identified across these *Camellia* genomes. The largest TPS repertoire was detected in *C. chekiangoleosa* (65 genes), whereas Yunkang10 contained the fewest (12 genes) (Figure 1, Table S2). The numbers of TPS genes identified in *C. taliensis*, *C. oleifera*, Shuchazao, Biyun, Huangdan, Tieguanyin, and Longjing43 were 51, 59, 33, 38, 36, 54, and 33, respectively. On average, wild species harbored ~58.3 TPS genes, while cultivated tea plants contained ~34.3 TPS genes; thus, wild species possessed 41.2% more TPS genes than cultivated accessions (24/58.3) (Figure 1, Table S2). These results indicate an apparent contraction of the TPS gene family, which may reduce terpene-related genetic diversity.

**Figure 2:**
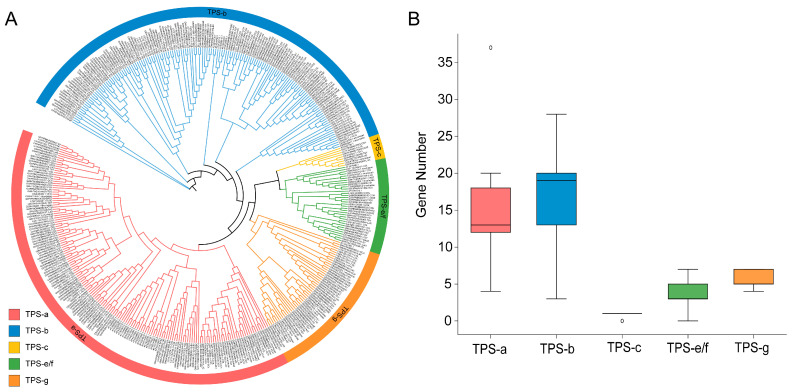
Phylogenetic analysis. (**A**) Phylogenetic tree constructed from TPS family genes of nine *Camellia* species and *A. thaliana* (the construction method is described in the Materials and Methods). (**B**) Box plot showing the distribution of gene numbers among TPS subfamilies.

Based on previous reports, we performed multiple sequence alignment and constructed a phylogenetic tree using 33 TPS proteins from *A. thaliana* together with the 381 *Camellia* TPS proteins (Figure 2A). According to tree topology and the established *Arabidopsis* subfamily classification, the TPS family was further divided into five subfamilies (TPS-a, TPS-b, TPS-c, TPS-e/f, and TPS-g). Among them, TPS-b was the largest subfamily with 149 members (an average of 16.6 genes per species), followed by TPS-a with 140 members (15.6 genes per species), whereas TPS-c was the smallest subfamily with only eight members (Figure 2A,B, Table S3). The pronounced differences in subfamily sizes suggest potential divergence in gene structures and functions among TPS subfamilies. Although the TPS family size varied substantially among *Camellia* species, most species retained members in all subfamilies; only Yunkang10 and Biyun lacked TPS-e/f and TPS-c members, respectively (Figure 2A,B, Table S3). Overall, despite considerable variation in TPS family size, the subfamily composition was largely conserved across *Camellia*, implying strong constraints imposed by core functions.

### 3.2. Gene Structure and Physicochemical Property Analysis of Tps Family Genes

**Figure 3:**
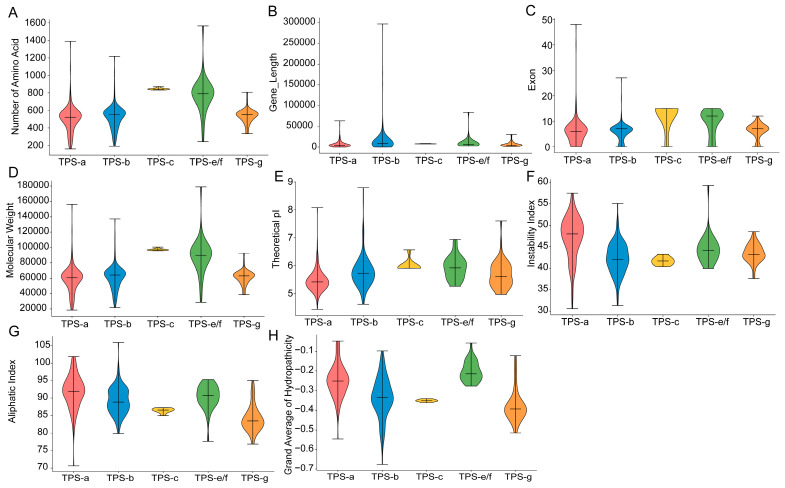
Physicochemical property analysis. (**A**) Distribution of amino acid length among TPS subfamilies. (**B**) Distribution of gene length among TPS subfamilies. (**C**) Distribution of intron number among TPS subfamilies. (**D**) Distribution of molecular weight among TPS subfamilies. (**E**) Distribution of theoretical isoelectric point (pI) among TPS subfamilies. (**F**) Distribution of instability index among TPS subfamilies. (**G**) Distribution of aliphatic index among TPS subfamilies. (**H**) Distribution of grand average of hydropathicity (GRAVY) among TPS subfamilies.

To clarify differences in TPS gene structures among subfamilies in *Camellia*, we summarized protein length (aa), gene length, intron length, and intron number for TPS family members from the nine *Camellia* genomes, and calculated the corresponding coefficients of variation (CVs) (Figure 3A–H, Tables S4 and S5). The results revealed pronounced inter-subfamily differences. The mean protein length was shortest in TPS-a (≈504.9 aa), intermediate in TPS-b/TPS-g (≈522.2/533.2 aa), and markedly longer in TPS-e/f and TPS-c (≈775.9/846.1 aa). The mean exon number was relatively low in TPS-b/TPS-a/TPS-g (≈5.66–6.16), increased in TPS-e/f (≈9.52), and was highest in TPS-c (≈12.38). The mean gene length was longest in TPS-b (≈14.72 kb), followed by TPS-e/f (≈10.41 kb), whereas TPS-a/TPS-g were shorter (≈6.93/6.98 kb), and TPS-c was intermediate (≈8.07 kb) (Figure 3A–H, Tables S4 and S5). More importantly, CVs showed clear differentiation: gene-length CV was extremely high in TPS-b (≈210.28%) and was also relatively high in TPS-e/f and TPS-a (≈135.25%/124.23%), but was very low in TPS-c (≈5.55%). Similarly, protein-length CV was higher in TPS-a/TPS-e/f/TPS-b (≈30.41%/27.06%/26.01%), whereas TPS-c again showed an extremely low CV (≈1.46%). Exon-number CV was highest in TPS-a (≈97.51%), while TPS-c still exhibited the lowest CV (≈43.82%) (Figure 3A–H, Tables S4 and S5). The consistently lowest CVs for gene length, protein length, and exon number in TPS-c indicate that the coding-region structure of TPS-c is highly conserved (Figure 3A–H, Tables S4 and S5). The CVs of gene length and exon number were far higher than those of protein length across subfamilies, suggesting that within-subfamily diversity is mainly driven by intron/structural variation.

Different subfamilies may display distinct physicochemical properties. Here, we predicted the molecular weight, theoretical pI, instability index, aliphatic index, and grand average of hydropathicity (GRAVY) for 381 TPS proteins (Figure 3A–H, Table S4). In terms of protein size, TPS-e/f showed the widest molecular-weight range and the highest mean (28.49–178.83 kDa, mean ≈89.0 kDa). Although TPS-a and TPS-b had similar means (≈58.4/60.5 kDa), both exhibited broad ranges (TPS-a: 18.53–156.28 kDa; TPS-b: 21.69–137.05 kDa), indicating substantial variation in sequence length/domain expansion within these subfamilies. In contrast, TPS-c displayed a highly concentrated MW distribution (95.72–100.60 kDa, mean ≈97.3 kDa), consistent with its more conserved structural features. Regarding pI, the overall distribution was predominantly acidic, with only 2.4% of genes showing pI > 7 (basic) (Figure 3A–H, Table S4). TPS-a/TPS-b had the broadest pI ranges (4.44–8.08 and 4.62–8.79, respectively), TPS-e/f ranged from 5.27 to 6.94, TPS-g from 4.97 to 7.60, and TPS-c was the most concentrated (5.91–6.57). For the instability index, most proteins were predicted to be unstable (85.8%), whereas ~14.2% were stable. For the aliphatic index, the maximum and minimum values were 105.94 and 70.62, respectively; the highest mean aliphatic index was observed in TPS-a (91.86), whereas the lowest mean was observed in TPS-g (86.46) (Figure 3A–H, Table S4). Overall, all GRAVY values were negative, indicating that TPS proteins in all subfamilies are generally hydrophilic. Among them, TPS-g and TPS-b were the most hydrophilic (GRAVY ≈ −0.515 to −0.122/−0.675 to −0.098; mean ≈ −0.377/−0.335), whereas TPS-e/f was relatively more “hydrophobic” (≈−0.277 to −0.058; mean ≈ −0.202) (Figure 3A–H, Table S4).

### 3.3. Core and Dispensable Tps Genes

**Figure 4:**
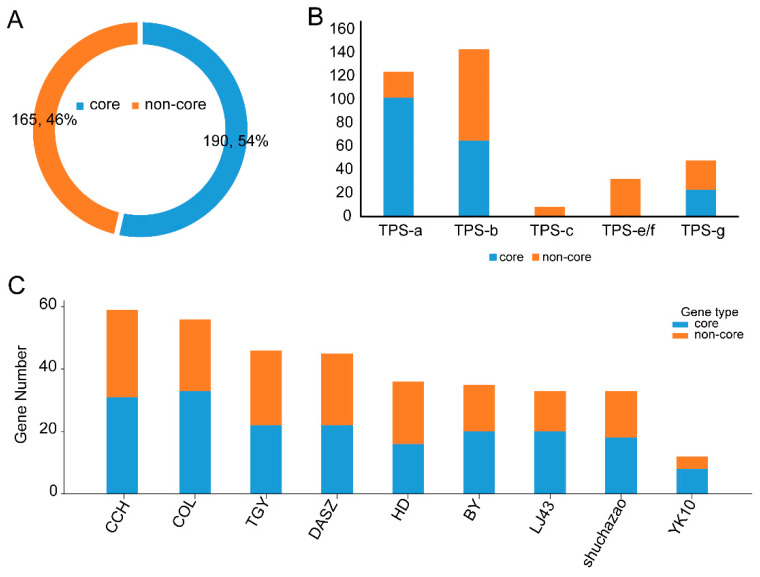
Core and non-core gene analysis. (**A**) Proportion of core and non-core genes. (**B**) Numbers of core and non-core genes in each subfamily. (**C**) Stacked bar chart of core and non-core genes across nine *Camellia* species.

To investigate TPS family genes in *Camellia* from a homology perspective, OrthoFinder was used to perform orthology clustering of the 381 TPS genes. In total, 355 genes were assigned to 19 orthogroups (OGGs) (Table S6), including 5 core orthogroups containing 190 genes (53.5%) and 14 dispensable orthogroups containing 165 genes (46.5%) (Figure 4A, Tables S6–S8). Based on the numbers of core and dispensable genes in cultivated versus wild tea species across the nine genomes, Mann–Whitney U tests were used to compare group differences for each metric, with Cliff’s delta as a nonparametric effect size. The results showed (Figure 4B, Tables S8–S10) that wild tea species had a higher total number of genes per species in core orthogroups than cultivated accessions (wild 32.33 ± 5.69 vs. cultivated 19.17 ± 6.40; U = 17.0, *p* = 0.0508, δ = 0.889, direction: wild > cultivated), and also had a higher total number of genes per species in non-core orthogroups (wild 21.00 ± 3.46 vs. cultivated 13.33 ± 5.47; U = 17.0, *p* = 0.0489, δ = 0.889) (Figure 4B, Tables S8–S10). However, the core proportion (core/total) was almost identical between the two groups (wild 0.60 ± 0.05 vs. cultivated 0.60 ± 0.07; U = 8.0, *p* = 0.8969, δ = −0.111), indicating that the difference mainly reflects “absolute copy-number scale” rather than the “composition ratio of core vs. non-core”. Furthermore, gene counts from all species were summarized into a 2 × 2 contingency table, and Fisher’s exact test showed no significant association between material type and core/non-core composition (OR = 1.071, *p* = 0.8279), supporting the conclusion that the proportional structure does not differ substantially (Figure 4B,C, Tables S8–S10).

From a statistical perspective, core/non-core status showed a significant association with subfamily. A chi-square test of independence based on a 5-subfamily × 2-status contingency table indicated that the two factors were not independent (χ^2^ = 93.90, df = 4, *p* = 1.95 × 10^−19^; total N = 355), with Cramér’s V = 0.542, suggesting a moderate-to-strong association (Figure 4B,C, Table S11). In addition, to mitigate potential impacts of small expected counts (e.g., expected values < 5 for TPS-c) on the chi-square approximation, a permutation test (20,000 permutations) was performed and also yielded a highly significant result (*p* ≈ 5.0 × 10^−5^), further supporting that the distribution of core/non-core status varies significantly among subfamilies. Subfamily-wise 2 × 2 Fisher tests with BH-FDR correction showed that TPS-a was significantly enriched for core genes (OR ≈ 9.91, FDR < 0.05; residual for core = +8.34), whereas TPS-e/f and TPS-c were significantly enriched for non-core genes/showed core depletion (core = 0; FDR < 0.05; residuals = −6.13 and −3.05, respectively) (Figure 4B,C, Table S12). TPS-b also showed relative core deficiency (OR ≈ 0.52, FDR < 0.05), while the association between TPS-g and core/non-core status was not significant (FDR > 0.05) (Figure 4B,C, Table S12). These results indicate that core and non-core genes are not randomly distributed across subfamilies but instead exhibit clear subfamily preferences.

### 3.4. Duplication Types and Cnv Analysis of the Tps Family

**Figure 5:**
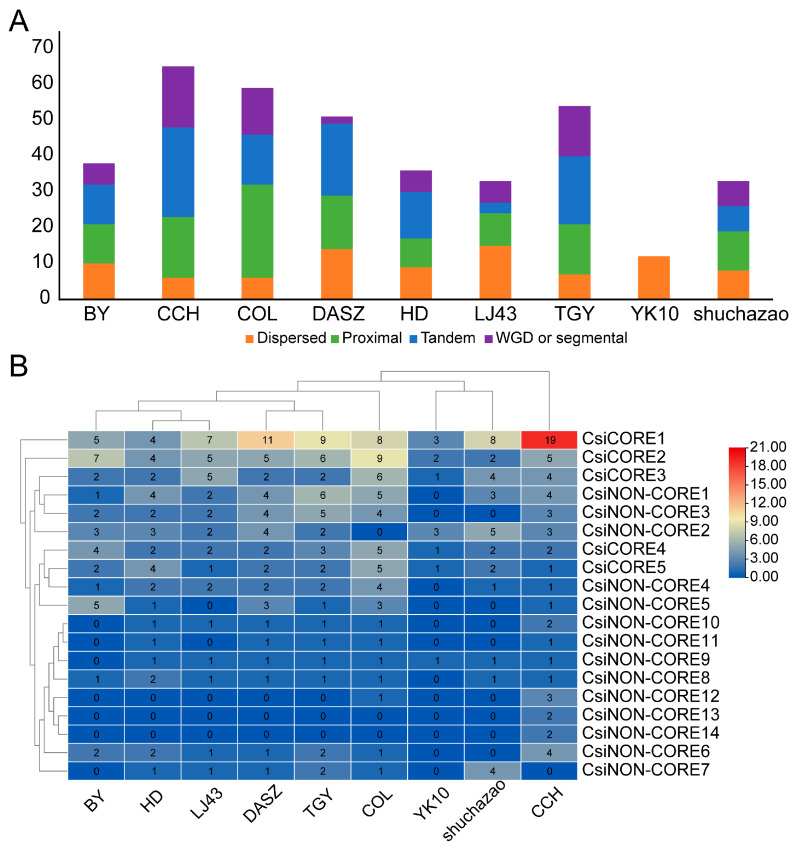
Gene duplication mode and CNV analysis. (**A**) Stacked bar chart showing the distribution of gene duplication modes across nine *Camellia* species. (**B**) Heatmap of copy number variation (CNV); orthogroups are shown on the *y*-axis, and species abbreviations are shown on the *x*-axis.

To elucidate the expansion mechanisms of the *Camellia* TPS family, we summarized gene duplication types in nine *Camellia* materials. Duplication types were assigned for all 381 TPS genes across the nine tea materials (Figure 5A, Table S13). Overall, TPS expansion was mainly derived from Tandem (112, 29.4%) and Proximal (111, 29.1%) duplications, followed by Dispersed (87, 22.8%), whereas WGD/segmental (71, 18.6%) contributed a relatively lower proportion; no Singleton-type genes were detected (Figure 5A, Table S13). Clear differences in duplication sources were observed among *Camellia* species: all TPS genes in YK10 were classified as Dispersed (12/12, 100%); Proximal accounted for the highest proportion in COL (26/59, 44.1%); and Tandem duplication was relatively high in CCH and DASZ (25/65, 38.5% and 20/51, 39.2%, respectively), with DASZ showing the lowest WGD/segmental proportion (2/51, 3.9%) (Figure 5A, Table S13).

Copy number variation (CNV) is considered a common form of structural variation in plants with pronounced functional consequences, which can influence phenotypes, domestication-related traits, and environmental adaptation by altering gene dosage and gene-cluster structure; in multi-species comparisons, “expansion–contraction” of gene families is often manifested through CNV [34]. Based on the OrthoFinder results for the TPS family across nine *Camellia* plants, all OGGs showed inconsistent copy numbers among species and thus exhibited CNV (Figure 5B, Table S6). Furthermore, among the five core orthogroups (CsiCORE1–CsiCORE5), copy numbers showed marked expansion differences: CsiCORE1 reached a maximum of 19 copies in CCH and also reached 11 copies in DASZ, whereas the highest copy numbers of CsiCORE2/3/4/5 were mostly observed in COL (Figure 5B, Table S6). In contrast, the 14 non-core orthogroups (CsiNON-CORE) showed strong insertion/deletion and copy-number fluctuations, as well as lineage-specific expansions; for example, CsiNON-CORE13/14 were detected only in CCH (2 copies each), whereas CsiNON-CORE1 reached up to 6 copies in TGY (Figure 5B, Table S6). These results indicate pronounced expansion/contraction divergence of the TPS family among *Camellia* species.

### 3.5. Selection Pressure Analysis of Tps Family Genes

**Figure 6:**
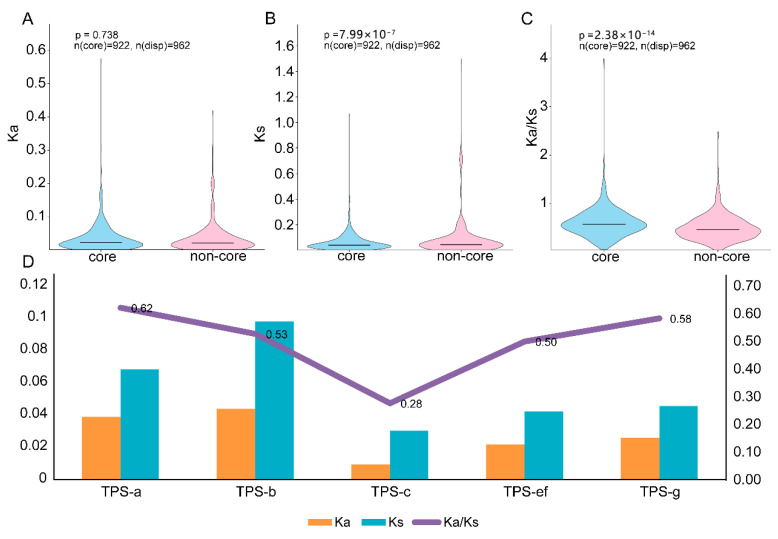
Selection pressure analysis. (**A**) Violin plot of Ka values for core and non-core genes (Student’s *t*-test). (**B**) Violin plot of Ks values for core and non-core genes (Student’s *t*-test). (**C**) Violin plot of Ka/Ks values for core and non-core genes (Student’s *t*-test). (**D**) Mean Ka, Ks, and Ka/Ks values across TPS subfamilies.

To further investigate selection pressures acting on the TPS family across the nine *Camellia* genomes, we calculated Ka, Ks, and Ka/Ks for all 1929 gene pairs derived from OGGs (Figure 6A–D, Table S14). The results showed that 1884 (97.67%) homologous pairs retained valid estimates, among which only 95 pairs (5.04%) had Ka/Ks > 1, whereas 1796 pairs (94.96%) had Ka/Ks < 1. The median Ka, Ks, and Ka/Ks values were 0.022, 0.043, and 0.516, respectively (Figure 6A–D, Table S14), indicating that the vast majority of TPS genes are subject to purifying selection. We further stratified homologous pairs by pan-genome gene type, including only pairs with consistent gene types at both ends (i.e., core–core and non-core–non-core). The median Ka, Ks, and Ka/Ks values for core pairs were 0.0223, 0.0402, and 0.5717, respectively, whereas those for non-core pairs were 0.0210, 0.0447, and 0.4522 (Figure 6A–D, Table S14). Except for Ka, the other two metrics differed significantly between the two groups (*t*-test, *p* < 0.05). These results suggest that core pairs exhibit more conserved neutral divergence, yet a higher proportion of nonsynonymous changes relative to synonymous changes, indicating divergent selection-pressure patterns between core and non-core genes.

We then calculated the mean Ka, Ks, and Ka/Ks values for homologous pairs within different TPS subfamilies across the nine *Camellia* species (Figure 6A–D, Table S14). Overall, the mean Ka/Ks values for all subfamilies were <1 (0.275–0.620), indicating that the *Camellia* TPS family is still predominantly under purifying selection at the sequence level, although the strength of constraint varies markedly among subfamilies. TPS-a showed the highest mean Ka/Ks (≈0.620; Ka ≈ 0.039, Ks ≈ 0.068), suggesting relatively weaker selective constraints and a potentially greater propensity for functional divergence. This was followed by TPS-g (Ka/Ks ≈ 0.582; Ka ≈ 0.026, Ks ≈ 0.046) and TPS-b (Ka/Ks ≈ 0.526), with TPS-b exhibiting the highest absolute substitution rates (Ka ≈ 0.044, Ks ≈ 0.098), indicating a faster overall evolutionary rate while still being dominated by purifying selection. In contrast, TPS-c showed the lowest mean Ka/Ks (≈0.275) as well as the lowest Ka and Ks values (Ka ≈ 0.009, Ks ≈ 0.030), indicating that this subfamily is the most conserved and is subject to stronger purifying selection constraints. TPS-e/f displayed an intermediate level (Ka/Ks ≈ 0.499) (Figure 6A–D, Table S14).

### 3.6. Pan-Transcriptome Analysis of the Tps Family

**Figure 7:**
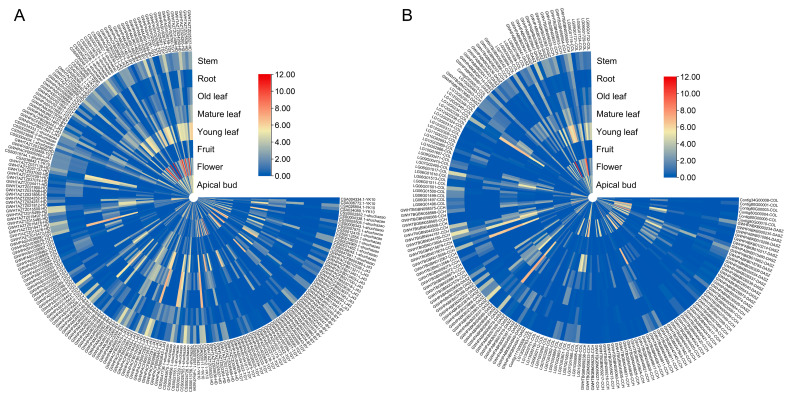
Transcriptome expression analysis across tissues in nine *Camellia* species. (**A**) Heatmap of transcriptomic expression levels across tissues in cultivated tea plants. (**B**) Heatmap of transcriptomic expression levels across tissues in wild tea plants.

To investigate differences in expression patterns of TPS family genes between wild and cultivated tea plants, we analyzed the expression levels of TPS genes in wild and cultivated tea across different tissues. The results showed that TPS family genes in cultivated tea exhibited higher expression distributions in most tissues, with the most pronounced differences concentrated in economically important tissues, namely Mature leaf and Stem. The median expression level in Mature leaf was 1.025 in cultivated tea and 0.111 in wild tea; in Stem, the median expression level was 0.978 in cultivated tea and 0.214 in wild tea (Figure 7A,B, Tables S15 and S16). These results indicate that cultivated tea has higher expression levels in stem and leaf tissues. Except for Flower, expression differences between wild and cultivated tea were significant in the other seven tissues (Mann–Whitney U, *p* < 0.05; Flower *p* ≈ 0.075) (Figure 7A,B, Tables S15 and S16).

We further compared the expression patterns of 381 *Camellia* TPS genes across five subfamilies (TPS-a/b/c/e/f/g) (Figure 7A,B, Tables S15 and S16). The results showed that TPS expression exhibited strong tissue preference and clear subfamily differentiation. TPS-g was the most strongly and broadly expressed subfamily, with median expression levels of 4.456, 4.052, and 2.952 in Young leaf, Apical bud, and Flower, respectively, suggesting high transcriptional activity across multiple tissues. TPS-e/f and TPS-b mainly peaked in Mature leaf and Stem (TPS-e/f: Mature leaf = 2.014, Stem = 1.595; TPS-b: Mature leaf = 1.021). TPS-a showed a peak in Flower (0.526), whereas TPS-c displayed an obvious Stem/Root preference (Stem = 1.967, Root = 1.173), indicating a concentrated and conserved expression pattern (Figure 7A,B, Tables S15 and S16).

## 4. Discussion

From a pangenome perspective, we systematically characterized the terpene synthase (TPS) family in the genus *Camellia*. We identified a total of 381 TPS members from nine *Camellia* genomes and integrated pan-transcriptome expression profiles across eight tissues, thereby providing multi-genome–scale resources and a framework for elucidating the genetic basis of terpene aroma in tea plants. Notably, wild materials contained significantly more TPS genes than cultivated materials (mean 58.3 vs. 34.3), consistent with lower TPS copy numbers in cultivated accessions than in the sampled wild relatives (Figure 1, Table S2). Because TPS genes can be clustered, differences in genome assembly contiguity and annotation pipelines among genomes—especially older, more fragmented assemblies—may collapse or split duplicated loci and thus under- or over-estimate TPS gene counts. Meanwhile, although cultivated materials generally harbored fewer copies, they displayed higher TPS expression distributions in most tissues—especially in mature leaves and stems—implying that domestication may have reshaped “dosage and regulation” rather than simply retaining more gene copies. Because the transcriptome matrices were compiled from public resources and may originate from different studies, sampling conditions, growth environments, and library preparation/sequencing protocols could confound cross-group comparisons; therefore, the observed higher TPS expression in cultivated accessions should be interpreted as suggestive and warrants validation under controlled, matched conditions.

Compared with previous single-reference TPS surveys in tea, our nine-genome, orthogroup-based framework provides pangenome-level insight by separating a conserved core module from a dispensable component showing pronounced PAV and lineage-specific CNV [34]. Integrating these OGG/CNV patterns with duplication mode, Ka/Ks, and expression further links structural variation to evolutionary constraint and potential functional divergence beyond what single genomes can resolve. Consistent with general principles from pangenome studies, a single reference genome often underestimates the true size of a gene family and fails to capture widespread presence/absence variation (PAV), copy number variation (CNV), and lineage-specific expansions. Recent reports emphasize that pangenomes enable improved dissection of the genetic architecture underlying crop domestication, ecological adaptation, and complex traits, and that structural variation (including CNV) can influence metabolic networks and trait evolution through changes in gene dosage and gene-cluster organization [20,35,36]. Therefore, our observation that wild tea plants harbor more TPS copies whereas cultivated tea plants show higher and broader expression is not contradictory: domestication bottlenecks and artificial selection could have contributed to variable copies and lineage-specific expansions of TPS, while transcriptional reinforcement in economically important tissues suggests regulatory rewiring in aroma-relevant tissues in cultivated tea materials (Figure 5B, Table S6).

At the subfamily level, *Camellia* TPS genes are dominated by TPS-b (149) and TPS-a (140), whereas TPS-c comprises only eight members and is highly conserved, indicating that branches is generally enriched for volatile terpenes constitute the majority of the TPS repertoire in *Camellia* and that functional diversification is characterized by a clearly “biased expansion” [37] (Figure 2A,B, Table S3). This pattern is comparable to findings in other plant TPS studies. For instance, TPS-a represents one of the largest functional clades in the tomato genome, accompanied by pronounced cluster organization and tissue-specific expression divergence; in eucalyptus, the TPS family is larger and physical clusters are denser, highlighting the importance of tandem/proximal duplication and cluster organization in generating volatile diversity [38,39].

Gene-structure and physicochemical analyses further showed that TPS-c exhibited the lowest coefficients of variation for gene length, protein length, and exon number, and also had the lowest Ka/Ks, indicating stronger purifying selection and tighter structural constraints on this branch (Figure 6A–C, Table S14). In contrast, TPS-b displayed an extremely high coefficient of variation for gene length, TPS-a showed the highest coefficient of variation for exon number, and TPS-a also had a higher mean Ka/Ks, suggesting that these branches may more readily expand product spectra and regulatory modes via intron/structural remodeling, accelerated sequence evolution, and potential functional divergence—resembling patterns reported for the bZIP family in Cucurbitaceae under a pan-genome framework [40].

Regarding core and dispensable genes, we assigned 355 TPS genes into 19 orthogroups, including five core orthogroups (190 genes, 53.5%) and 14 variable orthogroups (165 genes, 46.5%). We also found a significant association between core/non-core status and subfamily, indicating that different functional branches differ in their stability within the pangenome [41]. Analyses of duplication mechanisms and CNV further revealed the dynamic sources shaping the *Camellia* TPS family (Figure 4A–C, Tables S7 and S8). TPS expansion was mainly driven by tandem and proximal duplications (29.4% and 29.1%, respectively), whereas the contribution of WGD/segmental duplication was relatively low. Moreover, all orthogroups exhibited inconsistent copy numbers among species, indicating pervasive CNV and marked lineage-specific expansions (Figure 5A, Table S13). This agrees with reports in maize and potato showing prominent TPS (or related metabolic) cluster organization and tandem expansion, supporting the view that local duplication and dosage changes are major routes shaping volatile terpene biosynthetic capacity [42,43]. Selection-pressure analyses indicated that the TPS family is overall dominated by purifying selection (median Ka/Ks = 0.516; only a small proportion > 1) (Figure 6A–C, Table S14), but constraint strength is clearly heterogeneous across branches: TPS-a showed the highest mean Ka/Ks whereas TPS-c showed the lowest, suggesting that, while maintaining the stability of basic catalytic functions, certain branches may acquire novel substrate preferences or product profiles through limited acceleration of sequence evolution, thereby promoting chemical diversification of volatiles. These functional annotations are putative predictions based on phylogenetic placement and sequence features and require experimental validation in Camellia.

Overall, this study, for the first time, reveals the dynamic evolution of the *Camellia* TPS family under a pangenome framework, driven by domestication, local duplication, and CNV, and further characterizes tissue-level expression divergence of TPS genes across *Camellia*. These results provide candidate genes and molecular resources for tea aroma improvement and molecular breeding.

## 5. Conclusions

In this study, we performed a pan-genome–based characterization of the terpene synthase (TPS) gene family across nine *Camellia* genomes and integrated pan-transcriptome profiles across eight tissues. We identified 381 TPS genes in total and found that wild relatives harbored substantially more TPS members than cultivated accessions, supporting a putative contraction of the TPS repertoire. Phylogenetic analyses classified *Camellia* TPSs into five subfamilies, dominated by TPS-b and TPS-a, whereas TPS-c was rare and exhibited the most conserved gene structure and the strongest purifying selection signals. Orthogroup inference further resolved the pan-genome composition of TPSs into five core and fourteen dispensable orthogroups, revealing widespread presence/absence and copy-number variation across lineages. Duplication-mode analyses indicated that tandem and proximal duplications contributed most to TPS expansion, consistent with lineage-specific copy-number increases in several orthogroups. Finally, expression profiling showed that cultivated tea generally exhibited higher TPS expression in most tissues, particularly in mature leaves and stems, and TPS-g displayed broad and strong transcriptional activity. Collectively, these results provide a multi-genome resource for *Camellia* TPS genes and highlight how domestication-related divergence, local duplication, and CNV jointly shape terpene-related genetic diversity, offering candidate targets for functional validation and aroma-oriented molecular breeding.

## Data Availability

All data supporting the findings of this study are provided within the manuscript and its Supplementary Figures and Tables. No new raw sequencing data were generated in this study.

## Supplementary Materials

The following supporting information can be downloaded at: https://www.mdpi.com/article/10.3390/genes17010094/s1, Table S1. Genome information; Table S2. Number of TPS genes in 9 Genome; Table S3. Number of subfamily genes in 9 Genome; Table S4. Analyse of Physicochemical property in 9 Genome; Table S5. Analyse of CV in submfaily; Table S6. Number of genes in per OGGs; Table S7. Number of core genes and non-core genes in 9 genome; Table S8. Number of core genes and non-core genes in subfamily; Table S9. Mann–Whitney U; Table S10. Fisher’s exact test; Table S11. Pearson Chi-square (independence); Table S12. Fisher+FDR; Table S13. Analysis of gene duplication in 9 Genome; Table S14. Analysis of Ka, Ks and Ka/Ks; Table S15. Expression of TPS genes in 9 Genome; Table S16. Mann–Whitney U.
